# Potential Correlation between Microbial Diversity and Volatile Flavor Substances in a Novel Chinese-Style Sausage during Storage

**DOI:** 10.3390/foods12173190

**Published:** 2023-08-24

**Authors:** Hongfan Chen, Xinyue Kang, Xinyi Wang, Xinya Chen, Xin Nie, Lu Xiang, Dayu Liu, Zhiping Zhao

**Affiliations:** 1 College of Food and Biological Engineering, Chengdu University, Chengdu 610106, China; 2 College of Food Science and Technology, Sichuan Tourism University, Chengdu 610100, China; 3 School of Basic Medical Sciences, Chengdu Medical College, Chengdu 610500, China

**Keywords:** Chinese-style sausage, microbial diversity, volatile flavor substance, high-throughput sequencing, correlation analysis

## Abstract

A novel Chinese-style sausage with Chinese traditional fermented condiments used as additional ingredients is produced in this study. The aim of this study was to investigate the microbial community’s structure, the volatile flavor substances and their potential correlation in the novel Chinese sausage. High-throughput sequencing (HTS) and solid-phase microextraction-gas chromatography-mass spectrometry (GC-MS) were, respectively, used to analyze the microbial diversity and volatile flavor substances of the novel Chinese-style sausage during storage. The results showed that *Firmicutes*, *Proteobacteria* and *Actinobacteria* were the predominant bacterial genera, and *Hyphopichia* and *Candida* were the predominant fungal genera. A total of 88 volatile flavor substances were identified through GC-MS, among which 18 differential flavor compounds were screened (VIP > 1), which could be used as potential biomarkers to distinguish the novel sausages stored for different periods. *Lactobacillus* exhibited a significant negative correlation with 2,3-epoxy-4,4-dimethylpentane and acetoin and a significant positive correlation with 2-phenyl-2-butenal. *Hyphopichia* significantly positively correlated with ester. *Leuconostoc* significantly positively correlated with ethyl caprate, ethyl palmate, ethyl tetradecanoate and ethyl oleate while it negatively correlated with hexanal. This study provides a theoretical basis for revealing the flavor formation mechanisms and the screening of functional strains for improving the flavor quality of the novel Chinese-style sausage.

## 1. Introduction

Fermented sausage, a kind of fermented meat product, is popular with consumers because of its unique flavor, rich nutrition and long shelf life. During the fermentation, microorganisms metabolize the fat and protein into flavor precursors such as fatty acids, peptides and amino acids through lipases and proteases, which further decompose to form characteristic aroma substances of sausage [1]. The unique flavor of sausages is closely related to lactic acid bacteria (LAB) and yeast. It has been well reported that LAB are the dominant microorganisms during fermentation [2], which produce organic acids such as citric acid and lactic acid through the fermentation of carbohydrates and ensure product safety [3]. Moreover, LAB promote protein gelation to improve the textural characteristics of sausage [4]. Demeyer et al. [5] observed that exopeptidase in *Lactobacillus* could interact with aminopeptidase in muscle to promote the formation of free amino acids, thus contributing to flavor formation. Hu et al. [6] suggested that *Pediococcus pentosaceus*, *Lactobacillus (L.) brevis*, *L. curvatus* and *L. fermentum* could degrade sarcoplasmic proteins and thus produce flavor compounds and their precursors. *Staphylococcus* is an essential aroma-producing microorganism in fermented meat products [7], which can generate volatile flavor compounds through carbohydrate fermentation, amino acid metabolism, lipid oxidation and esterase-catalyzed reactions [8]. Moreover, *Staphylococci* in meat products also contribute to inhibiting lipid oxidation, improving nitrate reduction ability, and promoting color development [9]. Yeast, an important fungus in fermented meat products, can help enhance the aroma of meat products by metabolizing proteins and carbohydrates into organic acids, esters, and other flavor compounds [10]. On the other hand, yeast can degrade collagen and actin in meat and thus improve the texture [11].

*Douban* and *Douchi* are traditional Chinese fermented condiments, which are rich in various functional microorganisms such as *Bacillus*, *Bacteroides* and *Lactobacillus* [12,13]. *Douban*, *Douchi*, *Furu* and *Laozao* are made from broad beans, black beans, tofu and glutinous rice by anaerobic fermentation [14,15]. *Douban*, *Douchi*, *Furu* and *Laozao* are rich in various free amino acids, which play an important role in improving the flavor of sausage [16]. On the other hand, *Douban* and *Douchi* are rich in esters and pyrazine compounds [17], which can give sausages a strong sauce aroma. Furthermore, *Laozao* is rich in various flavor components such as esters and alcohols, which can effectively enrich the flavor of sausage [18]. In this study, unsterilized traditional Chinese fermented condiments such as *Douban*, *Douchi*, *Furu* and *Laozao* were used as additional ingredients to produce a novel Chinese-style sausage. Compared to traditional Chinese sausages, the novel sausage in this study had a higher microbial abundance at the initial fermentation stage, giving the sausage a more distinctive fermented flavor. However, the microbial community’s structure, the volatile flavor substances and their potential correlation remain unclear.

In this study, HTS and GC-MS were, respectively, used to analyze the microbial communities and volatile flavor substances of the novel Chinese-style sausage with different storage periods. Moreover, the potential correlation between core microorganisms and volatile flavor substances was investigated. The results of this study could provide a theoretical basis for improving the flavor quality of the novel Chinese-style fermented sausages.

## 2. Materials and Methods

### 2.1. Production of the Novel Chinese-Style Sausage

The pork meat and fat were purchased from Goldkinn Foods (Suining, China). The unsterilized traditional Chinese fermented condiments, *Douban*, *Laozao*, *Douchi* and *Furu,* were purchased from the local market. The recipe for the novel sausage list is in Table 1. *Douban*, *Laozao*, *Douchi* and *Furu* were mixed together and ground well. Then, the minced pork meat and fat (*m*/*m*, 7:3) were mixed well with ground *Douban*, *Laozao*, *Douchi* and *Furu* and salt and cured at 4 °C for 12 h. The mixture was filled into natural pig casings and naturally air-dried (average temperature of 5–11 °C, December) until its weight was reduced by 30%. The sausages were vacuum-packed and then stored at room temperature for 0, 7, 14, 30 and 60 days (average temperature of 4–10 °C, December and January), labeled D0, D7, D14, D30 and D60, respectively. Three batches of sausages were prepared. An equal amount of sausage from each batch was collected and well mixed for analysis.

### 2.2. GC-MS Analysis

An analysis of volatile flavor substance was performed using Agilent 7890B gas chromatography and an Agilent Model 5977 MSD series mass selective detector with a quadrupole mass analyzer (Agilent Technologies, Inc., Santa Clara, CA, USA). Three grams of sausage were placed in a 15 mL headspace flask. The pretreatment conditions for the sausage samples with a CTC automatic injector were as follows: heating box temperature of 50 °C, heating time of 45 min, sample extraction time of 20 min and analysis time of 5 min. The chromatographic conditions were as follows: HP-5MS UI column (30 m × 0.25 mm × 0.25 µm), pressure of 32.0 kPa, flow rate of 1.0 mL/min and carrier gas He. The injection temperature was 250 °C, the initial temperature was 40 °C, and holding lasted for 3 min. The temperature rose to 65 °C at 4 °C/min, which was held for 3 min, then increased to 105 °C at 3 °C/min, lasting 2 min, then rose to 165 °C at a ratio of 6 °C/min, and finally increased to 230 °C at a ratio of 12 °C/min. Mass spectrometry conditions were as follows: electron ionization source EI, electron energy of 70 eV, ion source temperature of 230 °C, quadrupole temperature of 150 °C, detector voltage of 350 V and mass scanning range (m/z) of 50–550. For qualitative analysis, the flavor data were retrieved and matched in the NIST14.L library of the instrument, and the substances with a matching degree of more than 80% were selected. Three parallel experiments were performed for every sample.

### 2.3. DNA Extraction and PCR Amplification and Sequencing

According to the manufacturer’s instructions, total microbial genomic DNA was extracted from sausages using the E.Z.N.A.^®^ soil DNA Kit (Omega Bio-tek, Norcross, GA, USA). The quality and concentration of DNA were determined by using 1.0% agarose gel electrophoresis and a NanoDrop^®^ ND-2000 spectrophotometer (Thermo Scientific Inc., Waltham, MA, USA). The genomic DNA was stored at −80 °C prior to further use. The V3-V4 region of the bacterial 16S rDNA gene was amplified with the primers 338F (5′-ACTCCTACGGGAGGCAGCAG-3′) and 806R (5′-GGACTACHVGGGTWTC TAAT-3’). The ITS1-ITS2 region was amplified by using the primers ITS1-F (5′-CTTGGTCATTTAGAGGAAGTAA-3′) and ITS2 (5′-GCTGCGTTCTTCATCGATGC-3′). Purified amplicons were pooled in equimolar amounts and paired-end sequenced on an Illumina MiSeq PE300 platform (Illumina, San Diego, CA, USA) according to the standard protocols of Majorbio Bio-Pharm Technology Co. Ltd. (Shanghai, China).

### 2.4. Data Processing

IBM SPSS version 24 (IBM Inc., Armonk, NY, USA) was used for data processing. PCA and PLS-DA were conducted using the software SIMCA 14.1 (Umetrics, Umea, Sweden). A one-way analysis of variance (ANOVA) was performed among the means using Turkey’s HSD to determine the differences in volatile flavor substances between sausages with different storage periods (*p* < 0.05). The results were expressed as the mean values ± standard errors. The correlation between the dominant microorganisms and volatile flavor substances was calculated by using Spearman correlation coefficients and visualized in R 4.2.1.

## 3. Results and Discussion

### 3.1. α-Diversity Analysis

**Figure 1:**
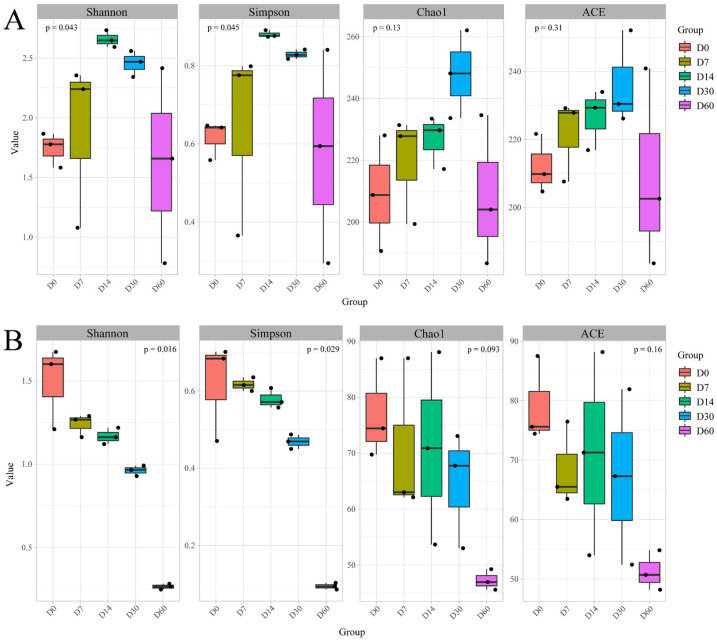
The α-diversity indices of bacteria (**A**) and fungi (**B**) in sausages with different storage periods.

The bacterial and fungal diversity of sausages at different storage periods was characterized by α-diversity indices such as Shannon, Simpson, Chao1, ACE and goods coverage, as shown in Figure 1 and Table S1. The Shannon and Simpson indices were often used to represent community diversity, with larger values indicating richer community diversity. The Chao1 and ACE indices were frequently employed to describe species richness, with higher scores indicating more species. In this study, the Shannon and Simpson indices of bacteria increased in the earlier storage stage, peaked at 14 days and then decreased continuously. The Chao1 and ACE indices of bacteria increased in the first 30 days of storage and then decreased, indicating that both the bacterial diversity and richness of sausages showed a trend of increasing first and then decreasing during the storage. Nonetheless, the Shannon, Simpson, Chao1 and ACE indices of fungi all displayed a decreasing trend, indicating that both fungal diversity and abundance of sausages tended to decrease during storage. In addition, the coverage index of all sausage samples was nearly 1.0, suggesting that the sequencing results could reflect the composition of bacterial and fungal communities in the sausage samples.

### 3.2. Analysis of Microbial Community Structure

**Figure 2:**
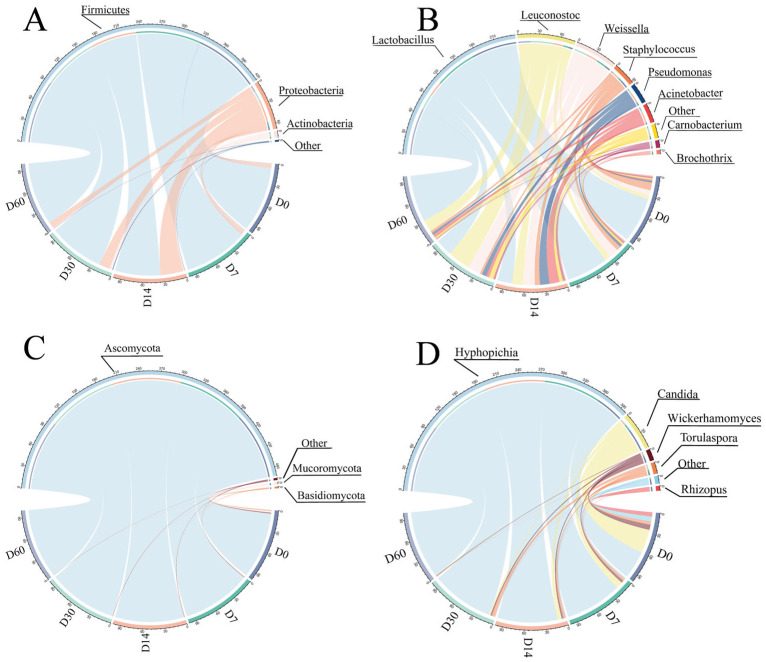
Relative abundance of bacteria at the phylum and genus levels (**A**,**B**) and fungi at the phylum and genus levels (**C**,**D**) of sausages at different storage periods.

The bacterial and fungal community structure of sausages with different storage periods was detected through Illumina MiSeq sequencing, as revealed in Figure 2. At the phylum level, Firmicutes was the most abundant bacterial phylum of sausage during storage, accounting for over 66%, with a maximum of 94.37%, followed by *Proteobacteria* and *Actinobacteria*. Firmicutes were the dominant phylum during sausage storage, consistent with a previous study [19]. Unno et al. [20] suggested that *Actinobacteria* were significantly correlated with the production of organic acids, free amino acids, ketones and sulfur-containing compounds in food. At the genus level, a total of eight core bacterial genera with relative abundance >1% [21] were identified, namely *Lactobacillus*, *Leuconostoc*, *Weissella*, *Staphylococcus*, *Pseudomonas*, *Acinetobacter*, *Carnobacterium* and *Brochothrix*. With the extension of storage time, bacterial diversity at the genus level gradually increased in the first 14 days and gradually decreased after 14 days, consistent with the α-diversity analysis. *Lactobacillus* was the dominant genus in sausages, which agreed with the results obtained by Yu et al. [22]. However, *Lactobacillus* and *Leuconostoc* exhibited an opposite growth trend during fermentation, most likely due to the antagonism and nutritional competition between the two microorganisms. LAB are considered the main bacteria in fermented sausage, which produce bacteriocin through fermentation and have an essential impact on the flavor of fermented products via participating in various biochemical pathways such as glycolysis and protein hydrolysis [23]. In addition, the abundance of *Staphylococcus* was lower than that of *Lactobacillus*, consistent with a previous study [7].

As for fungi, *Ascomycota* was the dominant phylum in sausages, with the relative abundance increasing continuously during storage. At the genus level, a total of five core fungal genera (relative abundance >1%) were identified, including four yeast genera (*Hypopichia*, *Candida*, *Wickerhamyces*, and *Torulaspora*) and one mold (*Rhizopus*). With the extension of storage time, *Hypophichia* gradually became the dominant fungal genus in sausages. However, the fungal diversity at the genus level continuously declined, consistent with the α-diversity. Yeast can grow on the surface of food and reduce the effect of oxygen on food quality [1]. Additionally, yeast could affect the flavor of meat products by hydrolyzing proteins and fats [24]. During storage, the relative abundance of *Hypopichia* continuously increased, whereas the abundance of other fungal genera decreased significantly. *Candida* plays a vital role in fermented food flavor by using different carbon sources for physiological metabolism to produce enzymes, acids and other byproducts [25].

### 3.3. Volatile Flavor Substances in the Fermented Sausages

**Figure 3:**
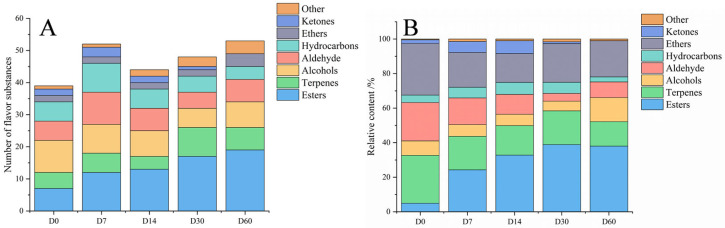
Types (**A**) and relative contents (**B**) of volatile flavor substances of the fermented sausages with different storage periods.

A total of 88 volatile flavor substances were identified through GC-MS, including 23 esters, 14 terpenoids, 13 alcohols, 13 aldehydes, 11 hydrocarbons, 4 ethers, 3 ketones and 7 other substances, as shown in Figure 3 and Table S2. The types of volatile flavor substances in sausages exhibited an initial increase followed by a decrease with the extension of storage. All types of volatile flavor substances changed significantly during storage. The esters increased continuously and accounted for approximately 38% after 30 days of storage. The alcohols, however, decreased during storage. Esters, terpenoids, alcohols, aldehydes and ethers were the primary volatile flavors during storage. In contrast to esters, aldehydes, ethers and terpenoids gradually decreased during storage.

Esters are considered the most abundant volatile flavor substances in sausage during storage, generally generated from non-enzymatic esterification reactions of alcohols and acids and enzymatic esterification reactions catalyzed by microorganisms [26]. Esters have a low threshold and are thus essential in meat products’ flavor. In this study, a total of 23 esters were detected, and 7, 12, 13, 17 and 19 esters were demonstrated in sausages stored for 0, 7, 14, 30 and 60 days with a relative content of 4.94%, 24.27%, 32.81%, 38.88% and 38.02%, respectively. The types and contents of esters increased significantly with the extension of storage, consistent with Sun et al’ research [27], which might be due to the continuous oxidative hydrolysis of fats during storage, leading to the production of alcohols and fatty acids, which were converted into esters by esterification. Esters are an essential source of fruity aromas in fermented sausages, such as ethyl butyrate with caramel and strawberry aromas [28].

Natural terpenoids and their derivatives have complex and varied aroma characteristics, with a low threshold and a rich aroma, which are an essential source of food flavor [29]. In this study, a total of 14 terpenoids were detected, and 6, 7, 4, 10 and 8 terpenoids were identified in sausages stored for 0, 7, 14, 30 and 60 days, with a relative content of 28.06%, 19.95%, 17.07%, 20.51% and 14.66%, respectively. D-limonene has a fresh orange aroma [28] and is the most abundant terpenoid in fermented sausages. The terpenoids decreased with the extension of storage, which was in line with a previous study [30] and might be due to the oxidation of terpenoids during storage [31]. On the other hand, microorganisms such as *Aspergillus niger* could catalyze the conversion of terpenes into alcohols and alkanes, resulting in a decrease in terpene [32].

Aldehydes are the most important flavor compounds with a low threshold and rich aroma, primarily derived from the oxidation of unsaturated fatty acids, such as oleic and linoleic acids, and the degradation of amino acids [33]. In this study, a total of 13 aldehydes were identified. Six, ten, seven, five and seven aldehydes were detected in sausages stored for 0, 7, 14, 30 and 60 days, with a relative content of 22.56%, 15.43%, 11.54%, 4.50% and 9.10%, respectively. The content of aldehydes decreased with the extension of storage, probably caused by the oxidation of aldehydes to acids [34]. The highest proportion of aldehydes was hexanal, mainly due to the oxidative decomposition of linoleic acid. However, hexanal is the primary source of the unpleasant flavor of fats and oils. In the present study, hexanal significantly decreased with the extension of storage, suggesting that appropriate storage was beneficial for reducing the unpleasant flavor of fermented sausages. The decrease in hexanal indicated that fat oxidation was inhibited, possibly by the spices added to the sausages [35]. On the other hand, some aldehydes were probably reduced to alcohols or oxidized to acids during storage [36]. Phenylacetaldehyde is a volatile flavor substance with a hawthorn odor generated from the metabolism of phenylalanine by microorganisms [27]. In this study, the phenylacetaldehyde increased, indicating that the degree of protein hydrolysis was increased as storage time expanded. Moreover, (E)-2-octenal, valeraldehyde, heptanal and heptanal were identified in sausages with fried, fatty, fruity and lemon aromas, respectively.

Alcohols are derived from multiple pathways, including carbohydrate fermentation, methyl ketone reduction, amino acid metabolism and lipid oxidation [37]. Saturated alcohols with higher thresholds have a limited influence on flavor [38], whereas unsaturated alcohols with lower thresholds strongly affect the flavor. 1-Octen-3-ol is primarily produced through linoleic acid oxidation, which has a strong aroma of mushrooms. The content of 1-Octen-3-ol in sausages decreased significantly during storage, indicating that the oxidation of fatty acids was inhibited during storage, which was consistent with the changes in aldehydes. In addition, terpin-4-ol with a pine flavor and terpene α-terpinol with a fruity sweet flavor were detected in fermented sausages [39].

The ethers have a strong and pleasant aroma, mainly coming from spices. In this study, four ethers were detected, namely dimethyl ether, 2-ethylhexyl glycidyl ether, anethole and estragole. Anethole was the most abundant ether in sausages, with a sweet and moist aniseed aroma, which is an essential characteristic flavor substance of spices such as star anise and fennel [40]. The anethole concentration decreased in the earlier stage and increased in the latter. A study has shown that *Pseudomonas* can metabolize the anethole as the sole carbon source into aromatic compounds such as p-anisic acid and p-hydroxybenzoic acid [41].

### 3.4. Hierarchical Clustering Analysis for Volatile Flavor Substances

**Figure 4:**
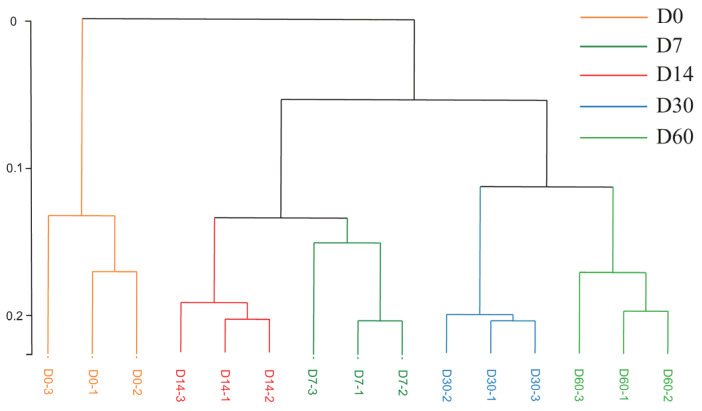
Hierarchical clustering analysis of volatile flavor substances of sausages stored for different periods.

To better understand the differences in the volatile flavor substances of the sausages, a hierarchical clustering analysis for volatile flavors was performed, as shown in Figure 4. The volatile flavor substances were clustered into three classes, where flavors for D0 sausage were clustered into one class alone, indicating that storage significantly changed the flavor of the fermented sausages. The flavors for D7 and D14 sausages were clustered into one class, and flavors for D30 and D60 sausages were clustered into one class.

### 3.5. PCA Analysis for Volatile Flavor Substances

**Figure 5:**
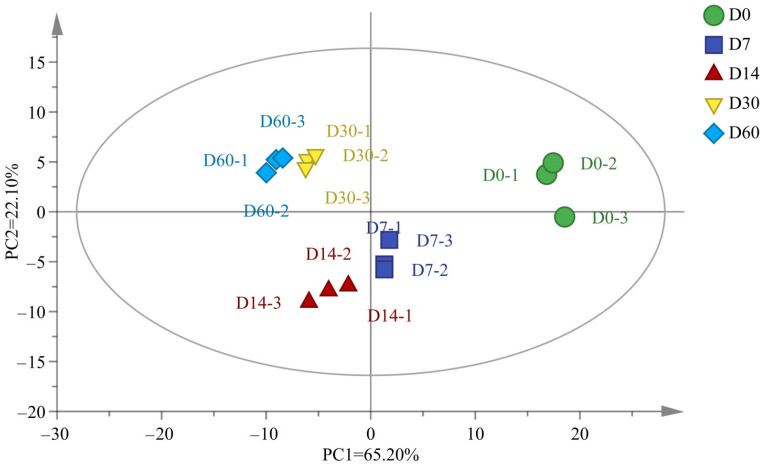
Scatter plot of principal component scores of sausages stored for different periods.

To further investigate the differences in volatile flavor substances of the fermented sausage during storage, PCA analysis was employed, as revealed in Figure 5. The PCA divided the flavor into two principal components, in which the contribution of PC1 was 65.20% and that of PC2 was 22.10%, with a cumulative contribution of 87.3%, indicating that the established PCA model could reflect the overall flavor of the sausages. R2X and Q2 are the most important parameters for evaluating the interpretability of the PCA model. In this study, the R2X = 0.874 > 0.5 and Q2 = 0.762, indicating that the PCA model was interpretable. The flavors in sausages stored for five different periods were completely separated and did not overlap. Furthermore, the 15 samples were distributed within the 95% confidence interval, suggesting that the volatile flavors of the sausages stored for five different periods differed significantly. The D7 and D14 sausages and the D30 and D60 sausages were located much closer, indicating that their flavor characteristics were more similar, which was consistent with the clustering analysis results.

### 3.6. PLS-DA Analysis

**Figure 6:**
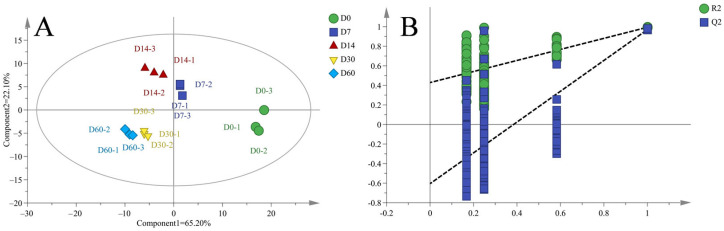
The PLS-DA scores (**A**) and permutation tests (**B**) for the volatile flavor substances of sausages stored for different periods.

The PLS-DA analysis was performed to further demonstrate the differences in volatile flavor substances in sausages, as shown in Figure 6A. The PLS-DA analysis divided the flavor into two principal components, in which the contribution of PC1 was 65.20% and that of PC2 was 22.10%, with a cumulative contribution of 87.3%, indicating that the PLS-DA model was reliable. The flavors in sausages were located in different areas of the coordinate system without overlap, suggesting that the flavor of each group was significantly different [42]. Moreover, the flavors for D7 and D14 sausages and D30 and D60 sausages were located much closer, indicating that their flavor characteristics were more similar, which was consistent with the cluster analysis described above. The 200-cycle iteration permutation test indicated that the established models were reliable, as shown in Figure 6B [43].

**Figure 7:**
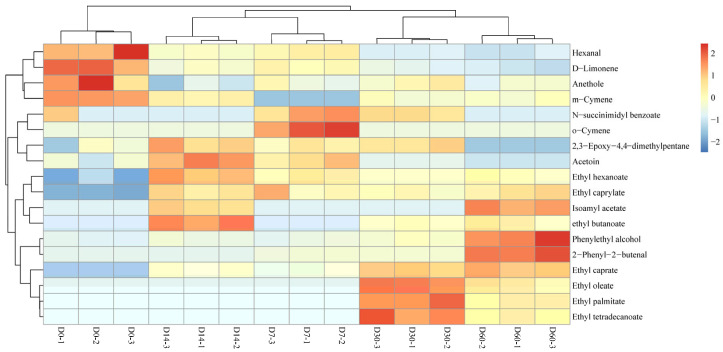
Hierarchical clustering analysis heat map of the main differential flavor substances in sausages stored for different periods.

The differential flavor substances of the sausages were screened based on the projected importance of variables (VIP) of PLS-DA. A total of 18 differential flavor substances were identified using VIP > 1 as the screening criterion [44], which could be used as the potential biomarkers to distinguish sausages stored for different periods, including hexanal, ethyl butanoate, anethole, ethyl hexanoate, ethyl caprylate and 13 others, as shown in Table 2. A hierarchical clustering analysis was performed to better investigate the differences between sausages with distinct flavors, as revealed in Figure 7. The D0 sausage contained a higher level of hexanal, D-limonene, anethole and m-cymene. The D7 sausage had higher amount of N-succinimidyl, benzoate and o-cymene. The D14 sausage had a high level of 2,3-epoxy-4,4-dimethylpentane, acetoin, ethyl hexanoate and ethyl caprylate. The D30 sausage possessed a higher content of ethyl caprate, ethyl oleate, ethyl palmitate, ethyl tetradecanoate and ethyl butanoate, while the D60 sausage was richer in isoamyl acetate, phenylethyl alcohol and 2-phenyl-2-butenal.

### 3.7. Key Volatile Flavor Substances in the Sausages Stored for Different Periods

The concentration and threshold of volatile compounds are the primary determinants of the overall flavor of meat products [45], and flavor compounds with high concentrations and low thresholds play an especially crucial role in food flavor. As seen in Table 3, a total of 15 flavor substances were detected by calculating the ROAV values (ROAV > 1). Ethyl caprylate, ethyl hexanoate, eucalyptol and linalool, with ROAV > 1 in all sausages, were the most critical volatile flavor substances in sausages, forming the basis of the characteristic flavor. Moreover, ethyl caprylate made the greatest contribution to the flavor of sausages. The ethyl caprylate, ethyl hexanoate and linalool have a fruity aroma, while eucalyptol has a lavender aroma. Furthermore, the nonanal, D-limonene, estragole, anethole, phenylacetaldehyde and 1-Octen-3-ol were the key volatile compounds in D0 sausage, which brought fruity, aniseed and mushroom aromas.

### 3.8. Potential Correlation between Microorganisms and Differential Volatile Flavor Substances

**Figure 8:**
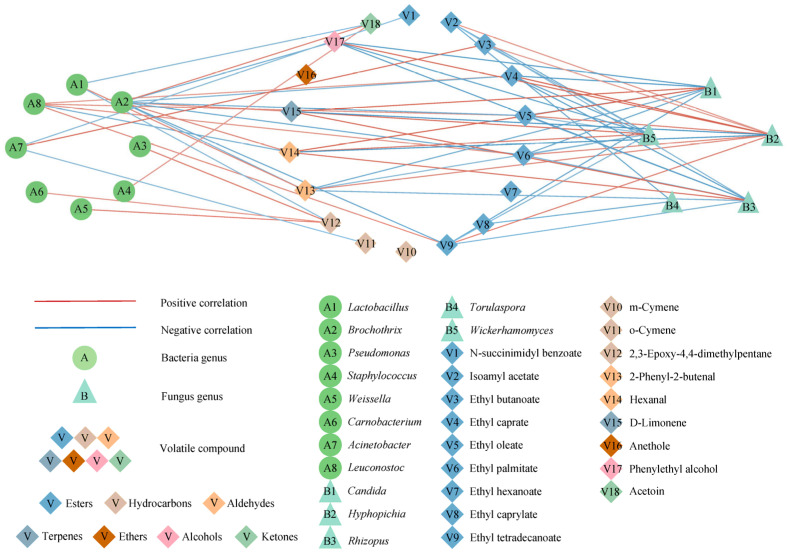
Potential correlation between dominant microorganisms and the differential volatile flavor substances.

It has been demonstrated that microorganisms play a crucial role in the flavor formation of fermented foods [46]. The correlation between the dominant microflora and differential volatile flavors in sausages stored for different periods was investigated by using Spearman correlation (|R| > 0.6, *p* < 0.05), as shown in Figure 8. *Lactobacillus* is considered to play an essential role in the formation of fruity and sour flavors in fermented foods [47]. In this study, *Lactobacillus* was significantly and negatively correlated with 2,3-epoxy-4,4-dimethylpentane (R = −0.602, *p* < 0.05) and acetoin (R = −0.631, *p* < 0.05), whereas it was correlated significantly positively with 2-phenyl-2-butenal (R = 0.706, *p* < 0.01), suggesting that *Lactobacillus* strongly decreased the fatty odor and increased fruity odor. *Staphylococcus* was significantly and positively correlated with acetoin (R = 0.618, *p* < 0.05), indicating that *Staphylococcus* greatly enhanced the grease aroma of sausage and exhibited an antagonistic effect with *Lactobacillus*, consistent with a previous study [48]. The *Acinetobacter* showed significantly negative correlations with o-cymene (R = −0.602, *p* < 0.05) and N-succinimidyl benzoate (R = −0.616, *p* < 0.05). *Leuconostoc* was significantly negatively correlated with hexanal (R = −0.711, *p* < 0.01) while displaying a positive correlation with ethyl caprate (R = 0.621, *p* < 0.05), ethyl palmitate (R = 0.665, *p* < 0.01), ethyl tetradecanoate (R = 0.693, *p* < 0.01) and ethyl oleate (R = 0.673, *p* < 0.01), suggesting that *Leuconostoc* was beneficial to the formation of fruity aroma in fermented sausages.

Compared to bacteria, fungi were more significantly correlated with flavors. Five fungal genera were identified as strongly correlated with sausage flavor formation. *Hyphopichia* was the dominant fungal genus during sausage storage, which significantly positively correlated with volatile flavors such as isoamyl acetate (R = 0.633, *p* < 0.05), ethyl butanoate (R = 0.609, *p* < 0.05), ethyl caprate (R = 0.939, *p* < 0.01), ethyl oleate (R = 0.774, *p* < 0.01), ethyl palmitate (R = 0.745, *p* < 0.01), ethyl tetradecanoate (R = 0.762, *p* < 0.01), 2-phenyl-2-butenal (R = 0.732, *p* < 0.01) and phenylethyl alcohol (R = 0.896, *p* < 0.01), agreeing well with Xu’s results [49]. *Rhizopus* is the common fungal genus in fermented foods with effective protein and fat hydrolysis ability [50]. It was unexpected that *Rhizopus* significantly negatively correlated with most of the esters in this study, inconsistent with a previous study [50].

## 4. Conclusions

In this study, a novel Chinese-style sausage is produced by using unsterilized Chinese traditional fermented condiments as auxiliary ingredients. A total of 88 volatile flavor substances were identified through GC-MS, including 23 esters, 14 terpenoids, 13 alcohols, 13 aldehydes, 11 hydrocarbons, 4 ethers, 3 ketones and 7 other substances. Eight predominant bacterial genera and five dominant fungal genera were identified by using HTS. The hierarchical clustering analysis and principal component analysis (PCA) results suggested that volatile flavor substances in sausages stored for different periods differed significantly. A total of 18 differential flavor substances were screened with VIP > 1, which could be used as potential biomarkers for distinguishing sausages stored for different periods. A total of 15 key flavor compounds were screened using ROAV values (ROAV > 1). It could be concluded that the microbial diversity closely correlated with the development of volatile flavor substances in the novel Chinese sausage based on Spearman correlation analysis (|R| > 0.6, *p* < 0.05). This study provides a theoretical basis for the screening and application of functional strains to improve the flavor quality of the novel Chinese-style sausage.

## Figures and Tables

**Table 1:** Recipe for preparation of the novel sausage.

Materials	*Douban*	*Laozao*	*Douchi*	*Furu*	Salt
Proportion of lean meat and fat	4%	3%	2%	1.5%	1%

**Table 2:** VIP values of the differential flavor substances in sausages stored for different periods.

Volatile Flavor Components	RT	CAS	VIP
Hexanal	6.071	66–25–1	2.80774
Ethyl butanoate	6.228	105–54–4	2.67423
Anethole	29.252	104–46–1	2.40669
Ethyl hexanoate	14.902	123–66–0	2.37757
Ethyl caprylate	25.189	106–32–1	2.19615
Acetoin	3.710	513–86–0	2.15824
D-limonene	16.003	5989–27–5	2.11602
Phenylethyl alcohol	20.281	60–12–8	1.93827
Ethyl caprate	32.685	110–38–3	1.89676
2,3-Epoxy–4,4-dimethylpentane	4.328	53897–30–6	1.67419
Isoamyl acetate	9.219	123–92–2	1.65980
N-succinimidyl benzoate	12.768	23405–15–4	1.37629
Ethyl tetradecanoate	38.560	124–06–1	1.33031
Ethyl palmitate	40.757	628–97–7	1.30464
m-Cymene	15.817	535–77–3	1.30454
o-Cymene	15.822	527–84–4	1.13554
2-Phenyl–2-butenal	28.605	4411–89–6	1.06599
Ethyl oleate	42.483	111–62–6	1.02648

**Table 3:** Volatile flavor odor activity values of flavors in sausages stored for different periods.

Volatile Flavor Components	CAS	Sensory Threshold mg/kg	ROAV				
			D0	D7	D14	D30	D60
Ethyl caprylate	106–32–1	0.000100	100	100	100	100	100
Ethyl hexanoate	123–66–0	0.000500	14.94	22.59	29.11	19.99	17.35
Eucalyptol	470–82–6	0.000260	48.68	3.99	3.28	2.65	2.58
Methional	3268–49–3	0.000040	-	-	-	9.26	16.17
Ethyl isovalerate	108–64–5	0.000200	-	4.88	5.79	3.45	3.29
Linalool	78–70–6	0.001500	11.99	1.34	1.35	1.36	1.10
ethyl butanoate	105–54–4	0.001000	-	-	4.34	1.91	2.29
Nonanal	124–19–6	0.003500	5.54	0.87	0.72	0.65	0.35
D-limonene	5989–27–5	0.034000	5.95	0.63	0.53	0.59	0.40
(E)–2-nonenal	18829–56–6	0.000065	-	4.75	3.37	-	-
Estragole	140–67–0	0.007500	5.22	0.50	0.43	0.65	0.51
Ethyl 2-methylbutanoate	7452–79–1	0.000150	-	1.90	2.55	-	0.96
Anethole	104–46–1	0.100000	2.11	0.22	0.17	0.30	0.20
Phenylacetaldehyde	122–78–1	0.009000	1.60	0.17	0.17	0.21	0.30
1-Octen–3-ol	3391–86–4	0.010000	1.09	0.14	0.09	0.06	-

## Data Availability

The data supporting the results of this study are included in the present article.

## Supplementary Materials

The following supporting information can be downloaded at: https://www.mdpi.com/article/10.3390/foods12173190/s1, Table S1: α-diversity index of new fermented sausage with different storage time; Table S2: Volatile flavor composition of new fermented sausages with different storage times.
