# Supplementary material for: Potential Correlation between Microbial Diversity and Volatile Flavor Substances in a Novel Chinese-Style Sausage during Storage
Source: Foods. 2023 Aug 24;12(17):3190. doi: 10.3390/foods12173190 (PMC10487076; doi:10.3390/foods12173190)
Supplement: Supplementary file 1 [file foods-12-03190-s001.zip › foods-2546844-supplementary.pdf]

Table S1. α-diversity index of the novel Chinese-style fermented sausage with different storage periods

	Bacteria					Fungus				
	Shannon	Simpson	Chao1	ACE	Coverage	Shannon	Simpson	Chao1	ACE	Coverage
D0	1.742	0.616	209.148	212.051	0.999	1.495	0.619	77.060	79.177	1.000
D7	1.891	0.646	219.518	221.558	1.000	1.239	0.617	70.704	68.469	1.000
D14	2.658	0.880	226.777	226.730	1.000	1.166	0.579	70.884	71.136	1.000
D30	2.456	0.829	247.950	236.241	1.000	0.961	0.469	64.603	67.194	1.000
D60	1.618	0.576	208.435	209.003	1.000	0.267	0.095	47.292	51.236	1.000

Table S2. Volatile flavor composition of the novel Chinese-style fermented sausages with different storage periods

NO.	Name and category of volatile flavor compounds		RT	CAS	Relative content/%				
					D0	D7	D14	D30	D60
A1	ethyl butanoate		6.23	105-54-4	-	-	3.63±0.32^{a}	1.24±0.14^{c}	1.90±0.09^{b}
A2	Ethyl lactate		6.71	97-64-3	-	0.50±0.14^{c}	0.66±0.05^{b}	0.56±0.06^{bc}	0.95±0.13^{a}
A3	Ethyl 2-methylbutanoate		8.07	7452-79-1	-	0.22±0.06^{ab}	0.32±0.04^{ab}	-	0.12±0.01^{bc}
A4	Ethyl isovalerate		8.20	108-64-5	-	0.77±0.22^{a}	0.97±0.15^{a}	0.45±0.05^{b}	0.55±0.06^{b}
A5	Isoamyl acetate		9.22	123-92-2	-	-	0.19±0.02^{b}	-	0.26±0.02^{a}
A6	vinyl hexanoate		14.13	3050-69-9	1.42±0.05^{a}	-	-	-	-
A7	linalyl formate		14.34	115-99-1	0.55±0.05^{a}	-	-	-	-
A8	Ethyl hexanoate		14.90	123-66-0	0.89±0.05^{d}	8.91±1.04^{b}	12.17±0.90^{a}	6.49±0.09^{c}	7.21±0.96^{bc}
A9	2-ethylhexyl butanoate		17.66	25415-84-3	-	-	-	-	0.14±0.02^{a}
A10	Ethyl benzoate		23.48	93-89-0	-	-	-	0.20±0.01^{a}	0.12±0.01^{b}
A11	Phenyl isothiocyanate		24.87	103-72-0	0.16±0.02^{ab}	0.31±0.01^{a}	0.25±0.02^{ab}	-	-
A12	Ethyl caprylate	Esters	25.19	106-32-1	1.20±0.20^{b}	7.89±0.13^{a}	8.36±0.86^{a}	6.50±0.60^{a}	8.31±1.00^{a}
A13	Ethyl phenylacetate		27.39	101-97-3	-	0.24±0.03^{b}	0.24±0.03^{b}	0.28±0.03^{b}	0.33±0.03^{a}
A14	Linalyl acetate		27.91	115-95-7	0.09±0.01^{d}	0.33±0.03^{bc}	0.28±0.03^{c}	0.43±0.04^{bc}	1.14±0.06^{a}
A15	Ethyl nonanoate		29.92	123-29-5	-	0.26±0.01^{c}	0.36±0.04^{b}	0.41±0.02^{a}	0.36±0.02^{b}
A16	9-decenoate ethyl		32.53	67233-91-4	-	0.40±0.04^{b}	0.36±0.02^{b}	0.82±0.09^{a}	0.86±0.04^{a}
A17	Ethyl caprate		32.69	110-38-3	0.62±0.05^{c}	4.18±0.52^{b}	5.02±0.48^{b}	9.61±0.45^{a}	10.18±0.75^{a}
A18	Ethyl laurate		35.90	106-33-2	-	0.26±0.02^{c}	-	1.61±0.13^{a}	1.07±0.02^{b}
A19	Ethyl tetradecanoate		38.56	124-06-1	-	-	-	3.22±0.50^{a}	1.38±0.15^{b}
A20	9-hexadecenoate ethyl		40.55	54546-22-4	-	-	-	0.66±0.08^{a}	0.31±0.07^{b}
A21	Ethyl palmitate		40.76	628-97-7	-	-	-	3.27±0.34^{a}	1.56±0.13^{b}
A22	ethyl linoleate		42.43	7619-08-1	-	-	-	0.52±0.06^{a}	-
A23	Ethyl Oleate		42.48	111-62-6	-	-	-	2.60±0.15^{a}	1.27±0.34^{b}
B1	Sabinene		13.35	3387-41-5	0.16±0.02^{a}	-	-	-	-
B2	β-Myrcene		14.35	123-35-3	0.81±0.08^{a}	-	-	0.91±0.08^{a}	-
B3	D-Limonene		16.00	5989-27-5	24.20±2.13^{a}	16.92±0.66^{b}	15.00±0.77^{b}	12.93±0.72^{c}	11.32±0.66^{c}
B4	Santolina triene	Terpenes	16.64	2153-66-4	-	-	-	-	0.06±0.10^{a}
B5	γ-Terpinene		17.51	99-85-4	0.56±0.10^{a}	0.30±0.06^{b}	0.35±0.03^{b}	0.31±0.03^{b}	-
B6	α-Copaene		32.14	3856-25-5	0.34±0.03^{b}	0.54±0.08^{b}	0.44±0.07^{b}	0.96±0.08^{a}	0.53±0.04^{b}

B7	Caryophyllene		33.07	87-44-5	1.98±0.09^{b}	1.69±0.16^{bc}	1.28±0.08^{c}	2.99±0.28^{a}	1.89±0.11^{b}
B8	cis-α-Bergamotene		33.40	18252-46-5	-	0.14±0.12^{b}	-	0.40±0.01^{a}	0.21±0.04^{b}
B9	α-caryophyllene		33.70	6753-98-6	-	-	-	0.25±0.01^{a}	-
B10	γ-Muurolene		34.38	30021-74-0	-	0.24±0.01^{a}	-	-	-
B11	(-)-α-Cedrene		34.38	469-61-4	-	-	-	-	0.24±0.21^{a}
B12	(+)-α-Funebrene		34.38	50894-66-1	-	-	-	0.83±0.07^{a}	0.16±0.27^{b}
B13	β-Bisabolene		34.58	495-61-4	-	-	-	0.40±0.05^{a}	-
B14	(+)-δ-cadinene		34.82	483-76-1	-	0.13±0.01^{c}	-	0.53±0.04^{a}	0.27±0.04^{b}
C1	3-methyl-1-Butanol		4.32	123-51-3	0.70±0.02^{b}	-	-	-	3.04±0.21^{a}
C2	3-methyl-1-Heptanol		4.47	1070-32-2	-	0.74±0.02^{a}	-	-	-
C3	(2R,3R)-(-)-2,3-Butanediol		5.84	24347-58-8	0.26±0.01^{b}	0.25±0.02^{b}	0.69±0.07^{b}	1.00±0.04^{b}	2.22±0.09^{a}
C4	1-Hexanol		8.97	111-27-3	0.23±0.09^{a}	-	-	-	0.23±0.02^{a}
C5	1-Octen-3-ol		13.96	3391-86-4	1.31±0.03^{a}	1.08±0.03^{ab}	0.73±0.08^{bc}	0.36±0.02^{c}	-
C6	Eucalyptol		16.13	470-82-6	1.52±0.11^{a}	0.82±0.07^{b}	0.71±0.05^{b}	0.45±0.05^{c}	0.56±0.06^{c}
C7	3,5-Octadien-2-ol	Alcohols	16.76	69668-82-2	-	0.18±0.01^{a}	-	-	-
C8	(Z)-2-Nonen-1-ol		18.34	41453-56-9	0.20±0.03^{a}	-	-	-	-
C9	trans-2-Pinanol		19.41	4948-29-2	-	-	0.38±0.04^{a}	-	-
C10	Linalool		19.68	78-70-6	2.15±0.21^{a}	1.59±0.11^{b}	1.69±0.16^{b}	1.33±0.16^{c}	1.38±0.10^{c}
C11	Phenylethyl Alcohol		20.28	60-12-8	1.06±0.10^{c}	1.42±0.26^{c}	1.59±0.17^{bc}	2.13±0.23^{b}	5.78±0.73^{a}
C12	Terpinen-4-ol		23.73	562-74-3	0.82±0.09^{a}	0.49±0.04^{b}	0.54±0.07^{b}	0.36±0.03^{c}	0.48±0.05^{b}
C13	α-Terpineol		24.59	98-55-5	0.11±0.01^{b}	0.26±0.01^{a}	0.21±0.01^{a}	-	0.25±0.02^{a}
D1	Valeraldehyde		3.44	110-62-3	-	0.24±0.01^{a}	-	-	-
D2	Hexanal		6.07	66-25-1	15.94±1.47^{a}	8.4±0.95^{b}	5.46±0.36^{b}	1.17±0.07^{c}	0.47±0.02^{c}
D3	Heptanal		10.15	111-71-7	1.12±0.02^{a}	0.95±0.15^{a}	0.84±0.09^{a}	-	0.96±0.08^{a}
D4	Methional		10.38	3268-49-3	-	-	-	0.24±0.02^{b}	0.54±0.07^{a}
D5	(Z)-2-Heptenal		12.68	57266-86-1	-	0.77±0.06^{b}	1.00±0.03^{a}	-	-
D6	(E)-2-Heptenal		12.69	18829-55-5	0.87±0.08^{a}	-	-	-	-
D7	Benzaldehyde	Aldehyde	12.78	100-52-7	-	-	-	-	1.60±0.14^{a}
D8	Phenylacetaldehyde		16.84	122-78-1	1.72±0.12^{b}	1.19±0.02^{c}	1.27±0.05^{c}	1.26±0.23^{c}	2.24±0.21^{a}
D9	(E)-2-Octenal		17.62	2548-87-0	0.28±0.04^{b}	0.82±0.07^{a}	0.70±0.07^{a}	-	-
D10	Nonanal		19.87	124-19-6	2.32±0.31^{a}	2.39±0.21^{a}	2.09±0.22^{a}	1.49±0.12^{b}	1.02±0.10^{b}
D11	(E)-2-Nonenal		22.82	18829-56-6	-	0.24±0.04^{a}	0.18±0.02^{b}	-	-
D12	(E)-2-Decenal		28.09	3913-81-3	-	0.12±0.01^{a}	-	-	-
D13	2-phenyl-2-butenal		28.61	4411-89-6	-	0.30±0.07^{b}	-	0.34±0^{b}	2.28±0.19^{a}
E1	2,3-Epoxy-4,4-dimethylpentane		4.33	53897-30-6	1.07±0.09^{c}	2.25±0.28^{b}	3.22±0.47^{a}	2.79±0.25^{ab}	-
E2	1-Chloropentane		5.24	543-59-9	0.21±0.36^{a}	-	-	-	-
E3	Octane		6.01	111-65-9	-	0.15±0.01^{a}	-	-	-
E4	p-Xylene		8.62	106-42-3	-	0.16±0.02^{a}	-	-	-
E5	Styrene	Hydrocarbons	9.54	100-42-5	0.55±0.05^{d}	0.62±0.8^{cd}	0.76±0.03^{bc}	0.94±0.11^{ab}	1.08±0.11^{a}
E6	1-Bromo-3,7-dimethyl-2,6-octadiene		14.35	35719-26-7	-	0.40±0.07^{b}	1.2±0.11^{a}	-	-
E7	m-cymene		15.82	535-77-3	1.80±0.05^{a}	-	1.11±0.05^{b}	0.81±0.12^{c}	0.83±0.08^{c}
E8	o-Cymene		15.82	527-84-4	-	1.10±0.13^{a}	-	-	-
E9	2,9-Dimethyl-5-decyne		18.34	19550-56-2	-	0.54±0.07^{a}	-	-	-
E10	2-Tetradecene		18.34	638-60-8	-	-	0.37±0.02^{a}	-	-

E11	α-Curcumene		34.18	644-30-4	0.29±0.06^{b}	0.38±0.05^{b}	0.28±0.04^{b}	0.91±0.01^{a}	0.45±0.04^{b}
F1	Dimethyl ether		6.94	115-10-6	-	-	-	-	1.07±0.07^{a}
F2	2-Ethylhexyl glycidyl ether	Ethers	17.66	2461-15-6	-	-	-	-	0.07±0.01^{a}
F3	Estragole		25.04	140-67-0	4.69±0.57^{a}	2.95±0.26^{b}	2.72±0.18^{b}	3.15±0.19^{b}	3.18±0.45^{b}
F4	Anethole		29.25	104-46-1	25.27±3.96^{a}	17.25±1.90^{bc}	13.94±1.96^{c}	19.28±1.68^{b}	16.62±1.54^{bc}
G1	Acetoin		3.71	513-86-0	1.35±0.17^{c}	5.06±1.19^{b}	7.10±0.88^{c}	1.03±0.07^{c}	-
G2	3,5-Octadien-2-one	Ketones	18.32	38284-27-4	-	0.86±0.04^{a}	-	-	-
G3	Fenchone		18.91	1195-79-5	0.74±0.09^{a}	0.42±0.05^{b}	0.36±0.05^{b}	-	-
H1	Diisopropylamine		3.47	108-18-9	-	-	0.76±0.07^{a}	-	-
H2	Methyltartronic acid		6.92	595-98-2	-	-	-	-	0.19±0.01^{a}
H3	N-succinimidyl benzoate		12.77	23405-15-4	0.41±0.02^{b}	1.38±0.02^{a}	-	1.08±0.09^{a}	-
H4	4-Ethyl-5-methylthiazole	Others	16.29	52414-91-2	-	-	-	-	0.28±0.03^{a}
H5	2,3-Dimethyl-5-ethylpyrazine		19.10	15707-34-3	-	-	-	0.25±0.03^{a}	0.28±0.03^{a}
H6	Octanoic acid		24.33	124-07-2	-	-	0.21±0.01^{a}	-	-
H7	Eugenol		31.79	97-53-0	-	-	-	0.24±0.02^{a}	0.22±0.01^{b}

Different superscript letters in the same row indicate significant differences (P<0.05).
